# Bis(1,3-thia­zol-2-aminium) hexa­chlorido­stannate(IV)

**DOI:** 10.1107/S1600536814014032

**Published:** 2014-06-21

**Authors:** Ruiting Xue, Lingmin Kong

**Affiliations:** a Zibo Environmental Protection Bureau, Shandong 255030, People’s Republic of China; b School of Marine Science and Technology, Zhejiang Ocean University, Zhoushan 316022, People’s Republic of China

**Keywords:** crystal structure

## Abstract

The asymmetric unit of the title compound, (C_3_H_5_N_2_S)_2_[SnCl_6_], contains one cation in a general position and one-half of the dianion situated on an inversion center. The geometry of the [SnCl_6_]^2−^ dianion is almost regular octa­hedral. In the crystal, weak N—H⋯Cl and N—H⋯S hydrogen bonds and electrostatic forces link cations and anions into a three-dimensional framework.

## Related literature   

For general background to inorganic-organic hybrid compounds, see: Zhang *et al.* (2009 ▶); Descazo *et al.* (2006 ▶); Li *et al.* (2007 ▶); Sanchez *et al.* (2005 ▶).
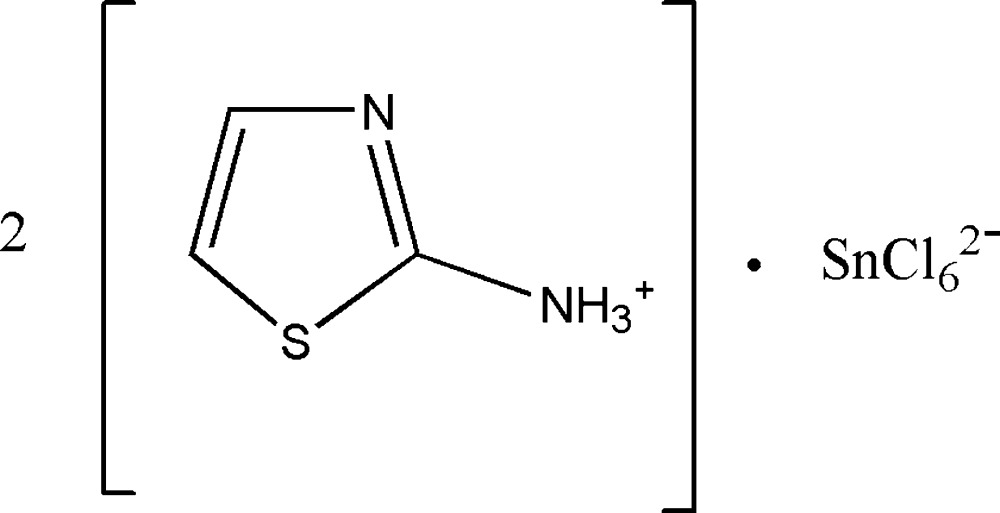


## Experimental   

### 

#### Crystal data  

(C_3_H_5_N_2_S)_2_[SnCl_6_]
*M* *_r_* = 533.69
Monoclinic, 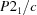

*a* = 7.9185 (7) Å
*b* = 8.6737 (10) Å
*c* = 12.8952 (14) Å
β = 101.629 (1)°
*V* = 867.50 (16) Å^3^
*Z* = 2
Mo *K*α radiation
μ = 2.63 mm^−1^
*T* = 298 K
0.43 × 0.41 × 0.40 mm

#### Data collection  

Bruker SMART CCD area-detector diffractometer
Absorption correction: multi-scan (*SADABS*; Sheldrick, 1996 ▶) *T*_min_ = 0.398, *T*_max_ = 0.420
4171 measured reflections
1529 independent reflections
1311 reflections with *I* > 2σ(*I*)
*R*_int_ = 0.039

#### Refinement  

*R*[*F* ^2^ > 2σ(*F* ^2^)] = 0.029
*wR*(*F* ^2^) = 0.082
*S* = 1.01
1529 reflections
89 parameters
H-atom parameters constrained
Δρ_max_ = 0.81 e Å^−3^
Δρ_min_ = −0.52 e Å^−3^

### 

Data collection: *SMART* (Bruker, 2007 ▶); cell refinement: *SAINT* (Bruker, 2007 ▶); data reduction: *SAINT*; program(s) used to solve structure: *SHELXS97* (Sheldrick, 2008 ▶); program(s) used to refine structure: *SHELXL97* (Sheldrick, 2008 ▶); molecular graphics: *SHELXTL* (Sheldrick, 2008 ▶); software used to prepare material for publication: *SHELXTL*.

## Supplementary Material

Crystal structure: contains datablock(s) global, I. DOI: 10.1107/S1600536814014032/cv5462sup1.cif

Structure factors: contains datablock(s) I. DOI: 10.1107/S1600536814014032/cv5462Isup2.hkl

CCDC reference: 796768

Additional supporting information:  crystallographic information; 3D view; checkCIF report

## Figures and Tables

**Table 1:** Hydrogen-bond geometry (Å, °)

*D*—H⋯*A*	*D*—H	H⋯*A*	*D*⋯*A*	*D*—H⋯*A*
N2—H2*A*⋯Cl1	0.89	2.70	3.522 (4)	155
N2—H2*C*⋯Cl3^i^	0.89	2.78	3.337 (4)	122
N2—H2*B*⋯Cl2^ii^	0.89	2.50	3.353 (4)	162
N2—H2*C*⋯S1^iii^	0.89	2.84	3.595 (4)	144

Symmetry codes: (i) 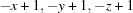; (ii) 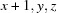; (iii) 
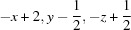
.

**Fig. 1.**
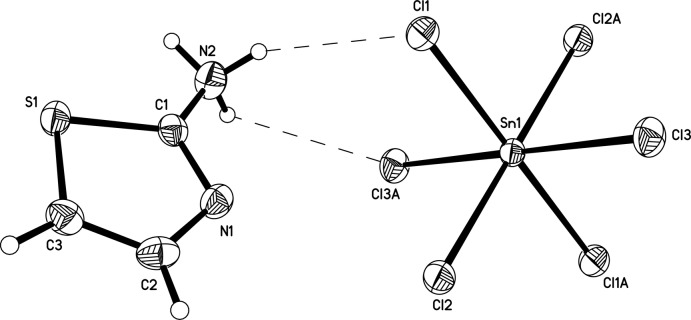
View of the title compound, showing 30% probability displacement ellipsoids and the atom-numbering scheme [symmetry code: (A) -x + 1, -y + 1, -z + 1]. Dashed lines denote weak N—H···Cl hydrogen bonds.

## supplementary crystallographic information

### S1. Comment

Considerable attention has been devoted to inorganic-organic hybrid materials over recent years·[Zhang *et al.*, 2009]. The supramolecular chemistry, the optical properties and the applications of this kind of hybrid materials have been reviewed in the literatures [Descazo *et al.*, 2006; Li *et al.*, 2007; Sanchez *et al.*, 2005]. Recently, we have prepared the title compound and here its crystal structure is reported.

This title compound contains SnCl6 inorganic anions and organic cations. The SnCl6 inorganic anion adopts an octahedron geometry, with average Sn—Cl distance 2.4278 Å. The inorganic anion and organic cation are linked through N—H···Cl hydrogen bond.

In the crystal structure, intermolecular N—H···Cl and N—H···S hydrogen bonds link cations and anions into a three-dimensioal framework. There are π-π stacking interactions involving the two thiazole rings, with a centroid···centroid distance of 3.769 (3) Å.

### S2. Experimental

2-aminothiazole (10 mmol) was dissolved to acid methanol solution (10 ml). Ten minutes later, an methanol solution (10 ml) of stannic chloride (5 mmol) was added with stirring. The mixture was stirred for 4 h. The solution was held at room temperature for about two weeks, whereupon yellow crystals suitable for X-ray diffraction analysis were obtained.

### S3. Refinement

All H-atoms were positioned geometrically and refined using a riding model, with C—H=0.93 Å (aromatic), N—H=0.89 Å (ammonium) and *U*_iso_(H) =1.2*U*_eq_(C), *U*_iso_(H) =1.5*U*_eq_(N)

### Crystal data

**Table:**

(C_3_H_5_N_2_S)_2_[SnCl_6_]	*F*(000) = 516
*M**_r_* = 533.69	*D*_x_ = 2.043 Mg m^−^^3^
Monoclinic, *P*2_1_/*c*	Mo *K*α radiation, λ = 0.71073 Å
*a* = 7.9185 (7) Å	Cell parameters from 2903 reflections
*b* = 8.6737 (10) Å	θ = 2.6–28.3°
*c* = 12.8952 (14) Å	µ = 2.63 mm^−^^1^
β = 101.629 (1)°	*T* = 298 K
*V* = 867.50 (16) Å^3^	Block, yellow
*Z* = 2	0.43 × 0.41 × 0.40 mm

### Data collection

**Table:**

Bruker SMART CCD area-detector diffractometer	1529 independent reflections
Radiation source: fine-focus sealed tube	1311 reflections with *I* > 2σ(*I*)
Graphite monochromator	*R*_int_ = 0.039
phi and ω scans	θ_max_ = 25.0°, θ_min_ = 2.6°
Absorption correction: multi-scan (*SADABS*; Sheldrick, 1996)	*h* = −9→8
*T*_min_ = 0.398, *T*_max_ = 0.420	*k* = −10→10
4171 measured reflections	*l* = −15→12

### Refinement

**Table:**

Refinement on *F*^2^	Primary atom site location: structure-invariant direct methods
Least-squares matrix: full	Secondary atom site location: difference Fourier map
*R*[*F*^2^ > 2σ(*F*^2^)] = 0.029	Hydrogen site location: inferred from neighbouring sites
*wR*(*F*^2^) = 0.082	H-atom parameters constrained
*S* = 1.01	*w* = 1/[σ^2^(*F*_o_^2^) + (0.0447*P*)^2^ + 0.6842*P*] where *P* = (*F*_o_^2^ + 2*F*_c_^2^)/3
1529 reflections	(Δ/σ)_max_ = 0.006
89 parameters	Δρ_max_ = 0.81 e Å^−^^3^
0 restraints	Δρ_min_ = −0.52 e Å^−^^3^

### Special details

**Table:**

Geometry. All e.s.d.'s (except the e.s.d. in the dihedral angle between two l.s. planes) are estimated using the full covariance matrix. The cell e.s.d.'s are taken into account individually in the estimation of e.s.d.'s in distances, angles and torsion angles; correlations between e.s.d.'s in cell parameters are only used when they are defined by crystal symmetry. An approximate (isotropic) treatment of cell e.s.d.'s is used for estimating e.s.d.'s involving l.s. planes.
Refinement. Refinement of *F*^2^ against ALL reflections. The weighted *R*-factor *wR* and goodness of fit *S* are based on *F*^2^, conventional *R*-factors *R* are based on *F*, with *F* set to zero for negative *F*^2^. The threshold expression of *F*^2^ > σ(*F*^2^) is used only for calculating *R*-factors(gt) *etc*. and is not relevant to the choice of reflections for refinement. *R*-factors based on *F*^2^ are statistically about twice as large as those based on *F*, and *R*- factors based on ALL data will be even larger.

### Fractional atomic coordinates and isotropic or equivalent isotropic displacement parameters (Å^2^)

**Table:**

	*x*	*y*	*z*	*U*_iso_*/*U*_eq_	
Sn1	0.5000	0.5000	0.5000	0.02834 (16)	
S1	1.05330 (13)	0.99651 (11)	0.36139 (10)	0.0395 (3)	
Cl1	0.76310 (12)	0.61913 (12)	0.59476 (8)	0.0430 (3)	
Cl2	0.40115 (11)	0.75120 (11)	0.42742 (9)	0.0422 (3)	
Cl3	0.36409 (13)	0.53966 (14)	0.65020 (9)	0.0438 (3)	
N1	0.7590 (4)	0.8875 (4)	0.3679 (3)	0.0398 (8)	
N2	0.9732 (4)	0.6990 (4)	0.3846 (3)	0.0474 (9)	
H2A	0.9521	0.6619	0.4451	0.071*	
H2B	1.0858	0.6943	0.3858	0.071*	
H2C	0.9167	0.6430	0.3308	0.071*	
C1	0.9217 (5)	0.8423 (5)	0.3731 (3)	0.0338 (9)	
C2	0.7362 (5)	1.0453 (6)	0.3548 (4)	0.0445 (10)	
H2	0.6303	1.0939	0.3503	0.053*	
C3	0.8803 (5)	1.1204 (5)	0.3493 (3)	0.0421 (10)	
H3	0.8870	1.2264	0.3400	0.051*	

### Atomic displacement parameters (Å^2^)

**Table:**

	*U*^11^	*U*^22^	*U*^33^	*U*^12^	*U*^13^	*U*^23^
Sn1	0.0269 (2)	0.0288 (3)	0.0304 (2)	0.00006 (13)	0.00865 (16)	0.00225 (14)
S1	0.0343 (5)	0.0390 (7)	0.0467 (7)	−0.0025 (4)	0.0117 (5)	0.0049 (5)
Cl1	0.0382 (5)	0.0456 (6)	0.0431 (6)	−0.0105 (4)	0.0028 (4)	0.0016 (5)
Cl2	0.0375 (5)	0.0338 (6)	0.0569 (7)	0.0046 (4)	0.0136 (4)	0.0113 (5)
Cl3	0.0439 (5)	0.0513 (6)	0.0408 (6)	0.0007 (5)	0.0192 (4)	−0.0030 (5)
N1	0.0332 (16)	0.048 (2)	0.0395 (19)	−0.0017 (15)	0.0110 (14)	−0.0024 (18)
N2	0.0449 (19)	0.045 (2)	0.056 (2)	−0.0028 (16)	0.0174 (17)	−0.0019 (19)
C1	0.038 (2)	0.038 (2)	0.028 (2)	−0.0007 (17)	0.0108 (16)	−0.0004 (18)
C2	0.040 (2)	0.053 (3)	0.040 (2)	0.012 (2)	0.0048 (18)	−0.003 (3)
C3	0.045 (2)	0.038 (2)	0.044 (2)	0.0062 (18)	0.0099 (19)	0.003 (2)

### Geometric parameters (Å, º)

**Table:**

Sn1—Cl3^i^	2.4224 (10)	N1—C2	1.387 (6)
Sn1—Cl3	2.4224 (10)	N2—C1	1.307 (5)
Sn1—Cl1	2.4238 (9)	N2—H2A	0.8900
Sn1—Cl1^i^	2.4238 (9)	N2—H2B	0.8900
Sn1—Cl2^i^	2.4374 (10)	N2—H2C	0.8900
Sn1—Cl2	2.4374 (10)	C2—C3	1.328 (6)
S1—C1	1.721 (4)	C2—H2	0.9300
S1—C3	1.723 (4)	C3—H3	0.9300
N1—C1	1.335 (5)		
			
Cl3^i^—Sn1—Cl3	180.0	C1—N1—C2	113.4 (3)
Cl3^i^—Sn1—Cl1	89.33 (4)	C1—N2—H2A	109.5
Cl3—Sn1—Cl1	90.67 (4)	C1—N2—H2B	109.5
Cl3^i^—Sn1—Cl1^i^	90.67 (4)	H2A—N2—H2B	109.5
Cl3—Sn1—Cl1^i^	89.33 (4)	C1—N2—H2C	109.5
Cl1—Sn1—Cl1^i^	180.0	H2A—N2—H2C	109.5
Cl3^i^—Sn1—Cl2^i^	91.15 (4)	H2B—N2—H2C	109.5
Cl3—Sn1—Cl2^i^	88.85 (4)	N2—C1—N1	124.1 (4)
Cl1—Sn1—Cl2^i^	90.61 (3)	N2—C1—S1	124.6 (3)
Cl1^i^—Sn1—Cl2^i^	89.39 (3)	N1—C1—S1	111.3 (3)
Cl3^i^—Sn1—Cl2	88.85 (4)	C3—C2—N1	113.5 (4)
Cl3—Sn1—Cl2	91.15 (4)	C3—C2—H2	123.3
Cl1—Sn1—Cl2	89.39 (3)	N1—C2—H2	123.3
Cl1^i^—Sn1—Cl2	90.61 (3)	C2—C3—S1	111.4 (4)
Cl2^i^—Sn1—Cl2	180.00 (5)	C2—C3—H3	124.3
C1—S1—C3	90.5 (2)	S1—C3—H3	124.3

Symmetry code: (i) −*x*+1, −*y*+1, −*z*+1.

### Hydrogen-bond geometry (Å, º)

**Table:**

*D*—H···*A*	*D*—H	H···*A*	*D*···*A*	*D*—H···*A*
N2—H2*A*···Cl1	0.89	2.70	3.522 (4)	155
N2—H2*C*···Cl3^i^	0.89	2.78	3.337 (4)	122
N2—H2*B*···Cl2^ii^	0.89	2.50	3.353 (4)	162
N2—H2*C*···S1^iii^	0.89	2.84	3.595 (4)	144

Symmetry codes: (i) −*x*+1, −*y*+1, −*z*+1; (ii) *x*+1, *y*, *z*; (iii) −*x*+2, *y*−1/2, −*z*+1/2.
